# P2X7R+CD8αβ^+^ T cells exacerbate postoperative ileus by inducing the loss of enterochromaffin cells

**DOI:** 10.1016/j.isci.2026.117491

**Published:** 2026-09-17

**Authors:** Fandi Bu, Shiyang Huang, Xiaobao Yang, Luyang Wei, Yingchi Yang, Zhongtao Zhang, Dong Zhang, Dan Tian

**Affiliations:** 1 Department of General Surgery, National Clinical Research Center for Digestive Disease, Beijing Friendship Hospital, Capital Medical University, Beijing 100050, China; 2 State Key Lab of Digestive Health, Beijing 100050, China; 3 Division of Hernia and Abdominal Wall Surgery, Department of General Surgery, Beijing Chao-Yang Hospital, Capital Medical University, Beijing 100020, China; 4 Beijing Key Laboratory of Organ Cultivation and Organ Protection in Transplantation, Beijing Friendship Hospital, Capital Medical University, Beijing 100050, China; 5 Medical Research Center, Beijing Institute of Respiratory Medicine and Beijing Chao-Yang Hospital, Capital Medical University, Beijing 100020, China; 6 Department of Gastroenterology, Beijing Chao-Yang Hospital, Capital Medical University, Beijing 100020, China; 7 Beijing Laboratory of Oral Health, Capital Medical University School of Basic Medicine, Beijing 100069, China

**Keywords:** postoperative ileus, CD8αβ^+^ intraepithelial cells, P2X7R, adenosine triphosphate, enterochromaffin cells

## Abstract

Postoperative ileus (POI) is a common complication after abdominal surgery, driven by inflammation. This study investigated how extracellular-ATP (eATP) and intestinal CD8αβ^+^ intraepithelial lymphocytes (IELs) contribute to POI. Using an intestinal manipulation mouse model, we found that CD8αβ^+^ IELs were increased and activated during POI, producing elevated granzyme B. Depletion of these T cells or inhibition of granzyme B ameliorated gastrointestinal transit impairment. Damage-induced eATP promoted granzyme B production in CD8αβ^+^ IELs via P2X7R signaling. Mechanistically, granzyme B degraded the tight-junction protein ZO-1, leading to enterochromaffin cell loss, reduced serotonin levels, and subsequent motility dysfunction. These findings reveal a pathway in which eATP activates CD8αβ^+^ IELs through the P2X7 receptor to drive enterochromaffin cell loss via granzyme B-mediated tight-junction degradation, identifying potential therapeutic targets for POI.

## Highlights

• CD8^+^ IELs were activated during POI
• eATP-P2X7R signaling upregulates GzmB in CD8^+^ T cells
• GzmB directly cleaves ZO-1, causing enterochromaffin cell loss
• ECC loss reduces 5-HT and impairs gastrointestinal motility

## Graphical abstract

**Figure:**
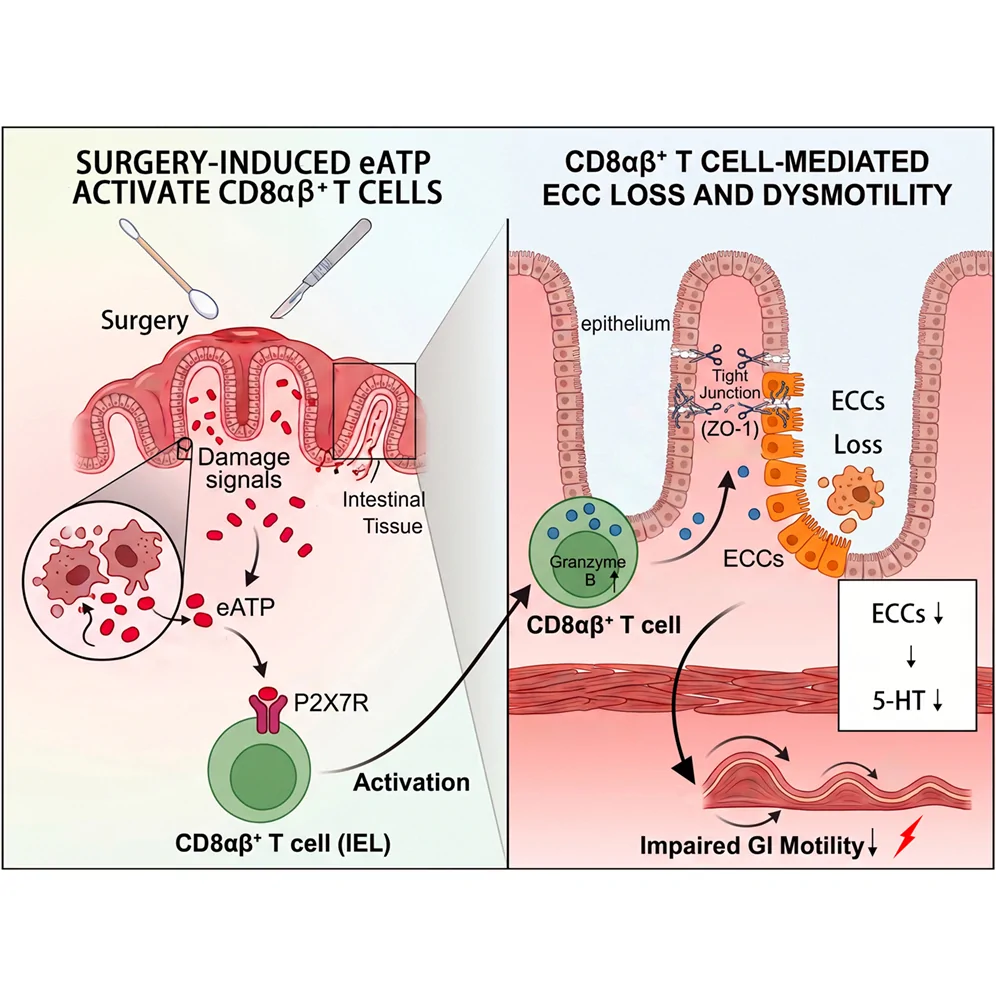

## Teaser

Surgery; Immunology; Gastroenterology

## Introduction

Postoperative ileus (POI) is a well-documented complication that frequently occurs after abdominal surgery. Patients with POI typically present with symptoms including nausea, abdominal bloating, and an inability to tolerate oral intake.^1^ The development of POI can be classified into two distinct phases: the early neurogenic phase and the subsequent late inflammatory phase. During the neurogenic phase, the inhibition of gastrointestinal (GI) motility is primarily mediated by the activation of adrenergic nerves resulting from surgical trauma. Notably, the stimulation of nociceptors and mechanoreceptors ceases upon abdominal closure. Following the neurogenic phase, the progression of POI is predominantly driven by an inflammatory response, which can persist for a duration of 2–4 days after surgery.

Intestinal immune cells are reported to play a pivotal role in the pathogenesis of POI. The involvement of myeloid cells in the pathogenesis of POI has been extensively investigated in numerous prior studies.^2^^,^^3^^,^^4^ Notably, a recent study has identified the presence of localized T cell aggregation within the duodenum of patients diagnosed with functional dyspepsia.^5^ Furthermore, the role of memory T helper 1 cells has been found to be crucial in driving the progression of POI.^6^ However, the precise mechanism underlying lymphocyte activation following mechanical stimulation in the context of POI remains unclear and warrants further investigation.

The surgical procedure, as an artificial form of trauma, unavoidably leads to tissue damage and the release of damage-associated molecular patterns (DAMPs), including nicotinamide adenine dinucleotide (NAD) and adenosine triphosphate (ATP).^7^ DAMPs can bind to pattern recognition receptors expressed on immune cells, initiating subsequent inflammatory cascades. Activation of various myeloid cells by DAMPs has been reported, resulting in the expression of inflammation-related genes, such as interleukin 6 (IL-6), cyclooxygenase-2 (COX2), and inducible nitric oxide synthase (iNOS).^8^ Previous studies have extensively investigated the effects of DAMPs on lymphocytes. Notably, high-mobility group box 1 (HMGB1) has been shown to regulate the aggregation of CD8^+^ T cells in the tumor microenvironment through a CXCL11-dependent mechanism.^9^

Extracellular-ATP (eATP), an important component of DAMPs, has garnered significant attention in recent years.^8^^,^^10^ Cell injury causes the accumulation of eATP in the tumor microenvironment.^11^ ATP triggers intracellular calcium influx in lymphocytes via the purinergic receptor P2 family. The ATP-P2X7R signaling pathway can also activate inflammasomes, thereby promoting local inflammatory responses.^12^^,^^13^ Moreover, it contributes to the secretion of IL-2 in activated T cells, although excessively high concentration of eATP can lead to lymphocyte death^14^ The sensitivity of various immune cells to eATP differs significantly,^14^^,^^15^^,^^16^^,^^17^ leaving many unresolved mysteries regarding the regulatory role of ATP in tissue immunity.

Enterochromaffin cells (ECCs) are a specialized type of epithelial cells responsible for regulating GI motility through the synthesis and secretion of serotonin.^18^^,^^19^ Tryptophan hydroxylase 1 (TPH1) serves as the rate-limiting enzyme in serotonin synthesis. Deficiency or dysfunction of TPH1 can result in inadequate serotonin levels, leading to significant impairment of GI motility.^20^^,^^21^^,^^22^ Despite the presence of motility disturbances in POI, the specific alterations in ECCs associated with POI have not been thoroughly investigated.

In this study, we observed a significant increase in CD8αβ^+^ intraepithelial lymphocytes (IELs) in mouse POI. Furthermore, these activated CD8αβ^+^ IELs exhibited an enhanced production of granzyme B, which exacerbated GI dysfunction in POI mice. Additionally, we discovered that eATP released as a result of tissue damage could promote the activation of CD8αβ^+^ IELs and the production of granzyme B via P2X7R signaling. Finally, we found that CD8αβ^+^ IELs in the small intestine contributed to a reduction in ECCs through the degradation of the tight junction by granzyme B, thereby further impairing GI transit.

## Results

### The increased proportion, activation, and granzyme B production of CD8αβ^+^ IELs are related to GI motility impairment in POI

**Figure 1:**
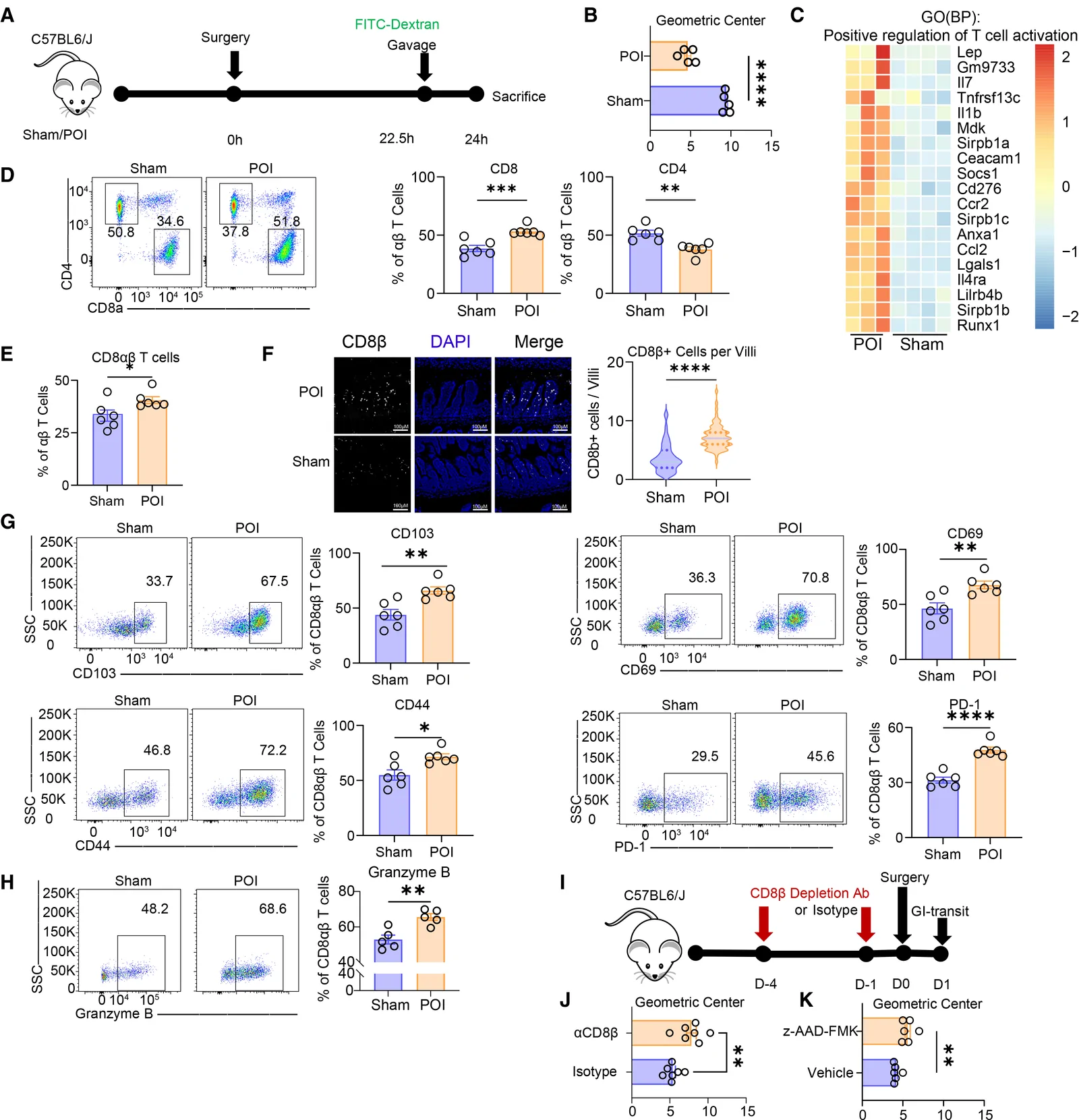
The increased proportion, activation, and granzyme B production of CD8αβ^+^ IELs are related to GI motility impairment in POI (A) Flowchart of intestinal manipulation surgery and GI-transit assay. (B) The GI transit was assessed by calculating the geometric center of FITC-dextran in the POI and sham groups. *n* = 5 mice. (C–E) (C) Gene expression differences of GO (BP): positive regulation of T cells activation pathway between POI and sham group. Proportional of (D) CD8^+^, CD4^+^ IELs, (*n* = 5 mice [Sham]; *n* = 6 mice [POI]) and (E) CD8αβ^+^ IELs in POI and sham mouse were assessed by flow cytometry. *n* = 6 mice. (F) The spatial distribution and proportion of CD8αβ^+^ IELs were assessed by immune fluorescence. *n* = 50 villis, 5 mice per group. Scale bars, 100 μm. (G) Expression of activation-associated markers (CD103, CD69, CD44, and PD-1) on CD8αβ^+^ IELs. *n* = 6 mice. (H) Granzyme B production of CD8αβ^+^ IELs in POI and Sham groups. *n* = 5 mice. (I) Flowchart of CD8αβ^+^ cell depletion assay. (J) Changes in GI transit after CD8αβ^+^ IEL depletion. *n* = 7 mice. Each point represents one replicate, and each experiment was repeated two times. ∗*p* < 0.05, ∗∗*p* < 0.01, ∗∗∗*p* < 0.001, or ∗∗∗∗*p* < 0.0001. Each column depicts the mean ± SEM.

We established a POI mouse model using intestinal manipulation (IM) and assessed GI-transit through gavage of FD70 (Figure 1A). The significant decrease in the geometric center of FD70 indicated impaired GI motility 24 h after surgery (Figure 1B). Subsequently, we investigated the transcriptional changes in the small intestine of POI mice. Principal component analysis (PCA) showed that the POI group and the sham group samples were divided into two clusters (Figure S1A). The volcano plot showed the most differentially expressed genes between POI and sham mouse intestines (Figure S1B). Gene Ontology (GO), Kyoto Encyclopedia of Genes and Genomes (KEGG), and gene set enrichment analysis (GSEA) revealed significant activation of immune cell regulation during the process of POI (Figures S1C and S1D). Several critical genes related to the T cell activation pathway, such as *Il7* and *Runx1*, were upregulated in the intestine of POI mice (Figure 1C).

To explore potential activated cells, we utilized flow cytometry to analyze IELs in POI mice. We observed a significant increase in the proportion of CD4^−^CD8^+^ IELs 12 h after surgery (Figure 1D). Among the CD8^+^ IELs, CD8αβ^+^ IELs also increased 12 h after surgery (Figures 1E and 1F). Regarding activation markers, we observed an upregulation in the expression of CD44, CD69, CD103, and PD-1 in CD8αβ^+^ IELs (Figure 1G). Granzyme B, a typical effector molecule of CD8^+^ T cells, also increased in CD8β^+^ IELs 12 h after surgery (Figure 1H).

We then investigated the role of CD8αβ^+^ IELs in POI through anti-CD8β antibody depletion before surgery (Figure 1I). As shown in Figure 1J, GI-transit demonstrated CD8αβ^+^ IEL depletion improved the GI-motility impairment. Additionally, we injected z-AAD-CMK (a small molecule inhibitor of granzyme B) after surgery, and the GI-transit assay showed that granzyme B aggravated the GI motility impairment (Figure 1K). These results collectively suggested that activated CD8αβ^+^ IELs and granzyme B production promoted the progression of POI.

### Increased eATP promotes the activation and granzyme B production of CD8αβ^+^ T cells via P2X7R during POI

**Figure 2:**
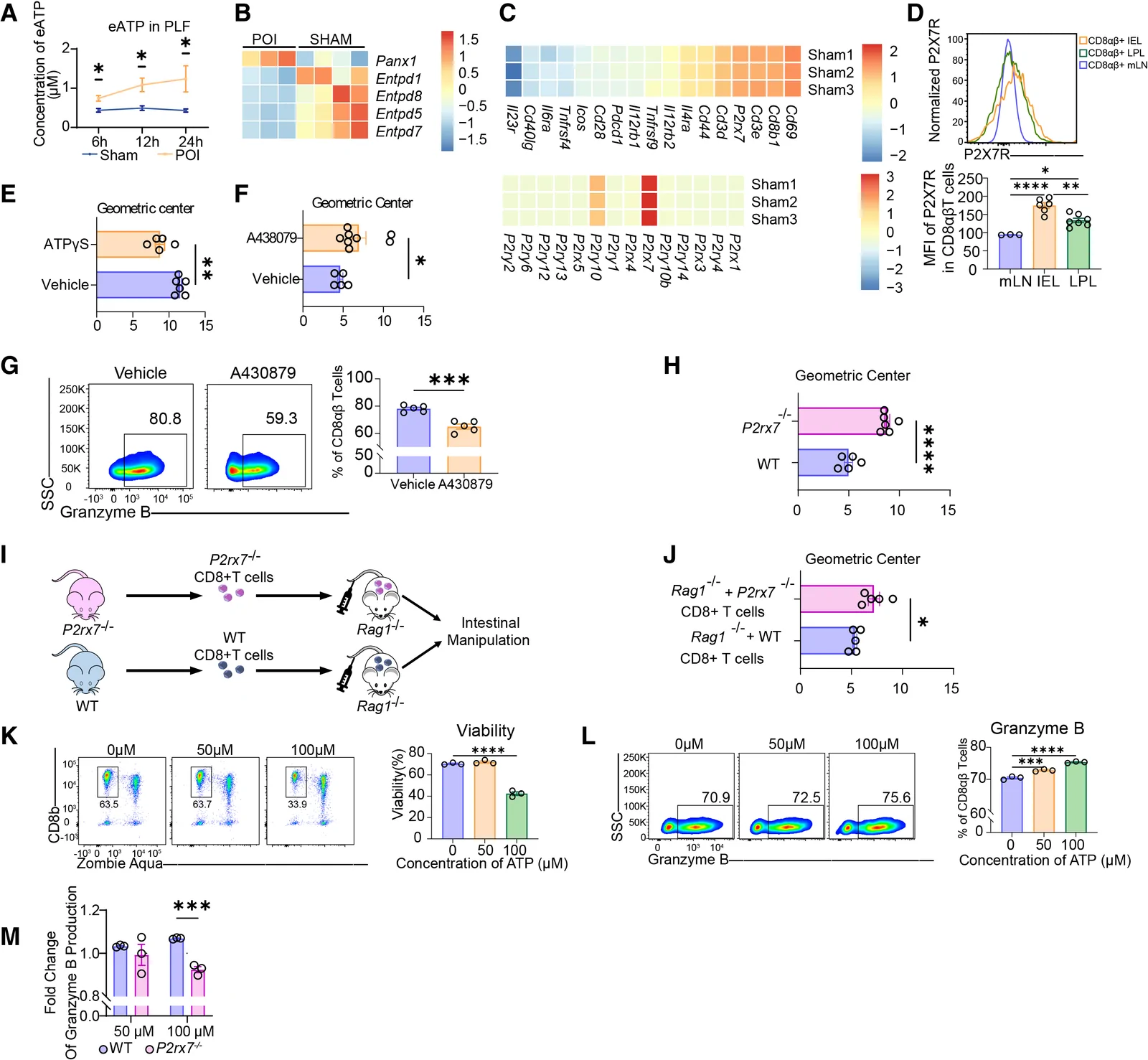
Increased eATP promotes the activation and granzyme B production of CD8αβ^+^ T cell via P2X7R during POI (A) PLF ATP concentration at 6, 12, and 24 h after surgery. *n* = 5 mice. (B) Differential expression of PANX1 and ENTPD1/5/7/8 after surgery. (C) Expression heatmap of surface marker and P2 receptor in intestinal CD8αβ^+^ T cells transcriptome data. (D) P2X7R expression of CD8αβ^+^ IELs in different tissue types. (E) Assessment of GI transit 3 h after intraperitoneal injection of ATP-γS. *n* = 5 mice (ATP-γS), *n* = 6 mice (vehicle). (F) GI transit of POI mice with and without intraperitoneal injection of A438079. *n* = 8 mice (A438079), *n* = 5 mice (vehicle). (G) Production of granzyme B in CD8αβ^+^ IELs with and without intraperitoneal injection of A438079 after surgery. *n* = 5 mice. (H) GI-transit 24 h after surgery in *P2rx7*^−/−^ and WT mice. *n* = 6 (*P2rx7*^−/−^), *n* = 5 (WT). (I) Flowchart of the adoptive transfer assay of CD8^+^ T cells and intestinal manipulation (IM) surgery. (J) GI transit 24 h after surgery in *Rag1*^−/−^ mice injected with *P2rx7*^−/−^ or WT CD8^+^, T cells. *n* = 5 mice. (K) Viability of CD8αβ^+^ IELs stimulated with ATP (0, 50, and 100 μm) for 30 min. *n* = 3 mice. (L) Granzyme B production of CD8αβ^+^ IELs stimulated with ATP (0, 50, and 100 μm) for 30 min. *n* = 3 mice. (M) Relative granzyme B production in CD8αβ^+^ IELs stimulated with ATP for 30 min in *P2rx7*^−/−^ and WT mice. *n* = 5 mice. ∗*p* < 0.05, ∗∗*p* < 0.01, ∗∗∗*p* < 0.001, or ∗∗∗∗*p* < 0.0001. Each column depicts the mean ± SEM.

Prior studies have noted heightened eATP levels after IM.^23^ Our research also detected significant eATP elevation at 6, 12, and 24 h after surgery in peritoneal lavage fluid (PLF) (Figure 2A). To explore the source of eATP, we investigated the ATP-related gene expression in small intestine. Significantly high expression *Panx1* (a large-pore ATP efflux channel gene) and the downregulation of *Entpd1*, *Entpd5*, *Entpd7*, and *Entpd8* (ectonucleoside triphosphate diphosphohydrolase genes) were observed in the POI intestine (Figure 2B). In addition, we observed that P2X7R (a well-established eATP receptor) exhibited high expression among both surface marker proteins of T cells and the P2R family in Sham mice (Figure 2C). Flow cytometry analysis indicated a notably higher expression of P2X7R on CD8αβ^+^ IELs compared to LPL or mLN (Figure 2D). To explore the role of ATP in the progression of POI, we intraperitoneally injected ATPγS (a non-hydrolytic analog of ATP) into healthy mice. Intriguingly, ATPγS induced a significant impairment of GI motility after 3 h of injection. (Figure 2E).

Subsequently, we investigated the potential involvement of P2X7R signaling in POI. To explore this, we administered A438079 (a small molecule inhibitor of P2X7R) via intraperitoneal injection following surgery. Notably, our results demonstrated that inhibiting P2X7R signaling with A438079 significantly alleviated GI motility impairment. (Figure 2F). Furthermore, the administration of A438079 reversed the elevated levels of granzyme B in CD8αβ^+^ IELs following IM (Figure 2G). To delve deeper into this phenomenon, we introduced *P2rx7*^−/−^ mice into our study. Remarkably, similar to the observations with A438079, the absence of P2X7R significantly ameliorated GI motility post-surgery (Figure 2H). To further investigate the role of *P2rx7* in CD8αβ^+^ T cell, we injected CD8αβ^+^ T cells from *P2rx7*^−/−^ or WT mice into *Rag1*^−/−^ immunodeficient mice for IEL reconstitution. The CD8αβ^+^ IELs could be reconstituted and showed no significant difference in count (Figure S2A). Two weeks later, all *Rag1*^−/−^ mice underwent IM (Figure 2I). Compared to WT CD8αβ^+^ T cells, adoptive transfer *P2rx7*^−/−^ CD8αβ^+^ T cells induced less GI motility impairment (Figure 2J). These data revealed that P2X7R expression on CD8^+^ IELs was sufficient to impair the GI-motility.

Subsequently, we investigated whether eATP directly affects CD8αβ^+^ IELs through P2X7R signaling. We isolated and stimulated CD8αβ^+^ IELs with ATP stimulation *in vitro*. Remarkably, we observed dose-dependent increases of cell death and granzyme B production, reinforcing the impact of eATP (Figures 2K and 2L). In contrast, *P2rx7*^−/−^ CD8αβ^+^ IELs exhibited no discernible response to eATP stimulation (Figure 2M). These data supported that heightened eATP levels activated CD8αβ^+^ IELs and subsequently enhanced granzyme B production via P2X7R.

### CD8αβ^+^ IELs induce the loss of ECCs and a decrease in 5-HT in POI

**Figure 3:**
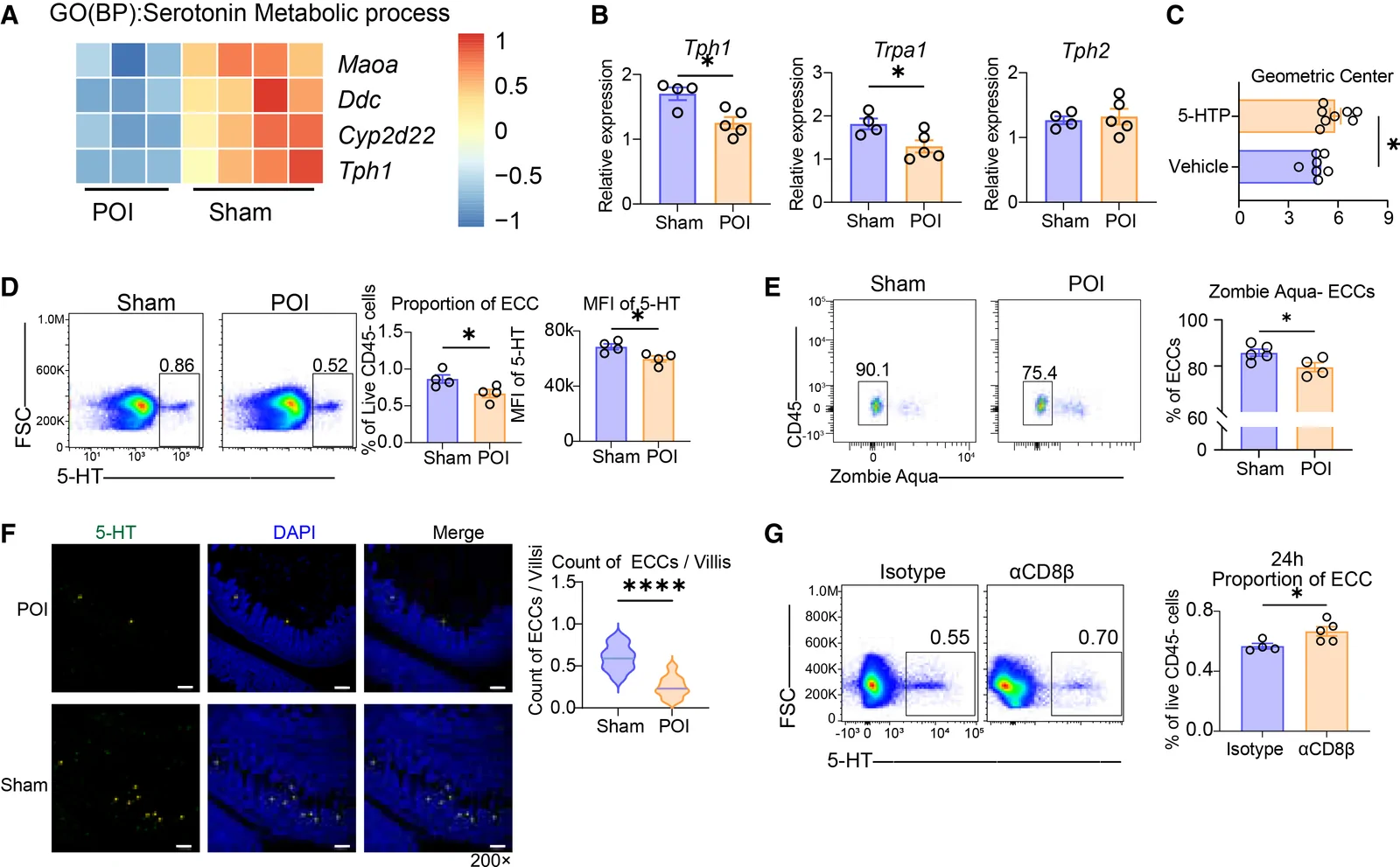
CD8αβ^+^ IELs induce the loss of ECCs and a decrease in 5-HT in POI (A) Differential gene expression of the Gene Ontology Biological Process term “serotonin metabolic process” between the POI and Sham groups. (B) qPCR analysis of *Tph1*, *Trpa1*, and *Tph2* expression in mice intestine. *n* = 4 mice (Sham) and *n* = 5 mice (POI). (C) Differences in GI-transit with or without 5-HTP administration via gavage in POI mice. *n* = 8 mice (5-HTP) and *n* = 7 mice (vehicle). (D) Flow cytometry analysis of ECC proportion and 5-HT content between the POI and Sham. *n* = 4 mice. (E) Flow cytometry analysis of Zombie Aqua- ECCs in the POI and Sham intestinal ECC cells. *n* = 5 mice (Sham) and *n* = 4 mice (POI). (F) Immune fluorescence analysis of ECCs count between the POI and Sham. Scale bars, 100 μm. (G) Flow cytometry analysis ECCs after CD8αβ^+^ IEL depletion. *n* = 4 mice (isotype) and *n* = 5 mice (αCD8β). ∗*p* < 0.05 or ∗∗∗∗*p* < 0.0001. Each column depicts the mean ± SEM.

The GO enrichment analysis of POI intestinal tissue indicated a significant decrease in the serotonin metabolic process pathway, with reduced expression of tryptophan hydroxylase 1 (*Tph1*), the rate-limiting enzyme in serotonin synthesis (Figure 3A). Downregulation of the central ECC genes *Tph1* and *Trpa1* (encoding transient receptor potential A1 expressed in ECCs), along with unchanged expression of *Tph2* (expressed in enteric neurons) confirmed ECC dysfunction (Figure 3B). Subsequently, we administered 5-hydroxytryptophan (5-HTP), a downstream metabolite of TPH1, to POI mice via gavage. The improved GI-motility after 5-HTP administration in POI mice indicated TPH1 insufficiency in POI (Figure 3C).

Flow cytometry data further revealed a decreased proportion of ECCs during POI, accompanied by a reduction in ECC viability (Figures 3D and 3E). However, the TPH2+ enteric neurons remained unaltered after POI (Figure S2B). Notably, our qPCR confirmed that TPH2+ enteric neurons were not reduced post-surgery (Figures 3B and 3D), ruling out direct neural ablation as the primary cause of early 5-HT deficiency. The cell counts of ECCs per villus decreased significantly after POI (Figure 3F). As the above data indicated the critical role of CD8αβ^+^ IELs in POI, we evaluated the impact of CD8αβ^+^ IELs on ECCs. Remarkably, depletion of CD8αβ^+^ IELs led to mitigation of ECC loss but not TPH2+ neurons (Figures 3G and S2C). Collectively, the data suggested that CD8αβ^+^ IELs contributed to ECC loss in POI.

### CD8αβ^+^ IELs promote tight-junction degradation and ECC loss via P2X7R-mediated granzyme B release

**Figure 4:**
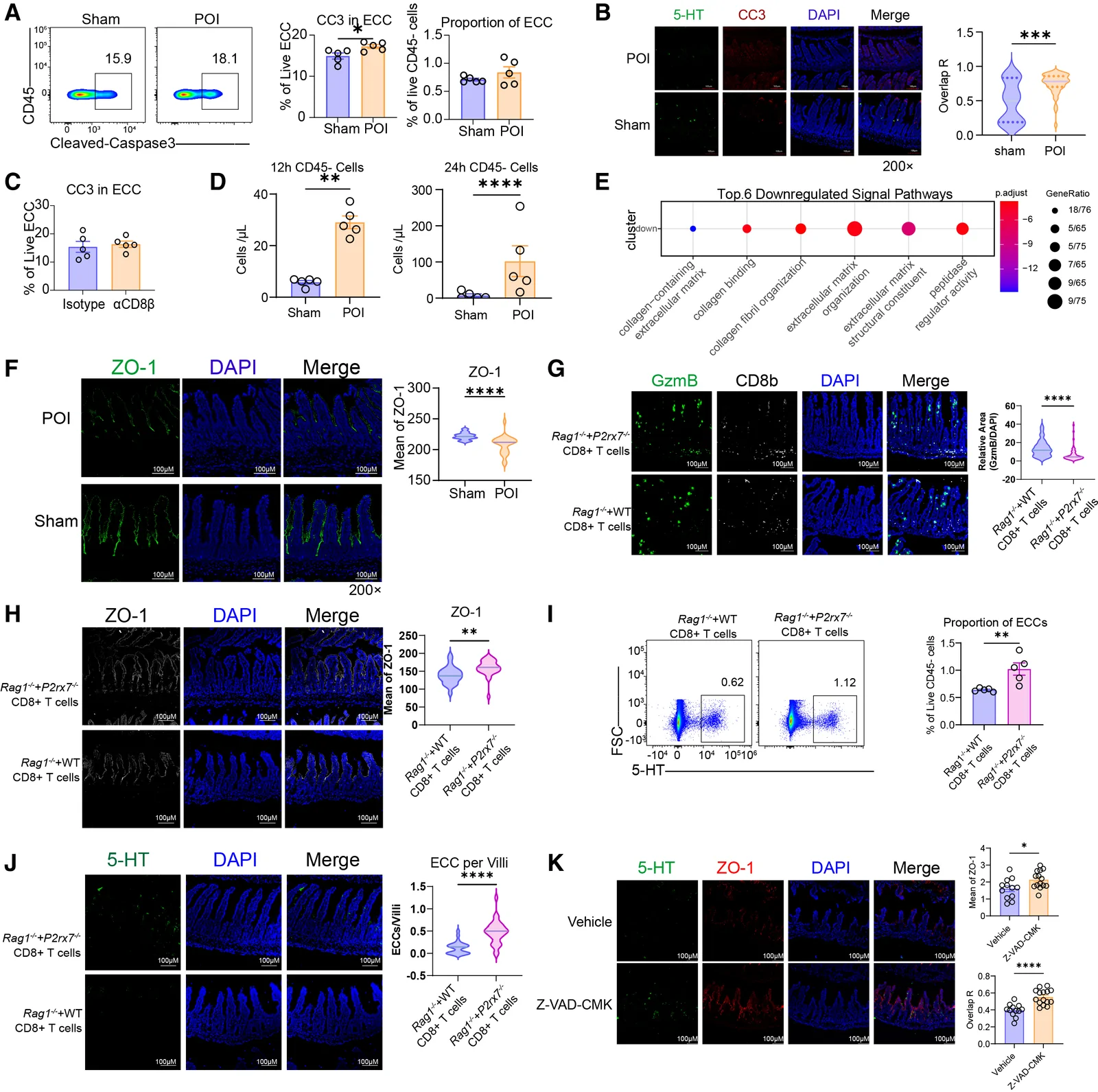
P2X7R signaling stimulates CD8αβ^+^ IELs, promoting ECC loss and tight junction degradation via granzyme B (A) Flow cytometry analysis of cleaved-caspase3 in ECCs and CD45^−^ epithelial cells. *n* = 5 mice. (B) Colocalization between CC3 and ECCs. *n* = 50 fields, 5 mice per group. (C)Proportion of ECCs after CD8β^+^ IEL depletion. *n* = 5 mice. Scale bars, 100 μm. (D) Changes in the count of CD45^−^ Cells in IELs of POI mice. *n* = 5 mice. (E)Top 6 downregulated signal pathways of GO enrichment in IELs 12 h after surgery. (F) The mean intensity of tight-junction protein ZO-1 in POI mice. *n* = 25 fields, 5 mice per group. Scale bars, 100 μm. *n* = 50 fields, 5 mice per group. (G)The mean area of granzyme B in CD8β^+^ IELs in *Rag1*^−/−^ mice following injection of *P2xr7*^−/−^ or WT CD8^+^ T cells. *n* = 50 Cells, 5 mice per group. Scale bars, 100 μm. (H) The mean of ZO-1 in *Rag1*^−/−^ mice following injection of *P2xr7*^−/−^ or WT CD8^+^ T cells. *n* = 25 fields, 5 mice per group. Scale bars, 100 μm. (I) Flow cytometry analysis of ECC proportion in *Rag1*^−/−^ mice following injection of *P2xr7*^−/−^ or WT CD8^+^ T cells. *n* = 5 mice. (J) Immunofluorescence analysis of ECC proportion in *Rag1*^−/−^ mice following injection of *P2xr7*^−/−^ or WT CD8^+^ T cells. *n* = 26 villis, 5 mice per group. Scale bars, 100 μm. (K) Immunofluorescence analysis of ZO-1 and colocalization of ECCs and ZO-1 following admission of granzyme B inhibitor Z-VAD-CMK. *n* = 12 fields (vehicle), *n* = 15 fields (Z-VAD-CMK), 5 mice per group. Scale bars, 100 μm ∗*p* < 0.05, ∗∗*p* < 0.01, ∗∗∗*p* < 0.001, or ∗∗∗∗*p* < 0.0001. Each column depicts the mean ± SEM.

Based on abovementioned findings, we proceeded to investigate how ECC loss occurs. Initially, we observed cleaved-caspase3 (CC3) in ECCs 12 h post-surgery. Both flow cytometry and IF analyses revealed increased CC3 preceding ECC loss during POI (Figures 4A and 4B). Our initial assumption was that CD8αβ^+^ IELs might induce ECC damage through cytotoxicity. However, depleting CD8αβ^+^ IELs did not reduce CC3 in ECCs 12 h post-surgery (Figure 4C).

Intestinal immune cells accumulate during POI. Interestingly, we observed a significant increase in CD45^−^ cells 12 and 24 h after surgery during the IEL isolation procedure (Figure 4D). This indicated that CD45^−^ intestinal cells in POI mice appeared more vulnerable to the IEL isolation procedure. Extracellular granzyme B has been reported to degrade the extracellular matrix.^24^ We propose that granzyme B degrades the extracellular matrix, promoting ECC loss. Transcriptome sequencing of epithelial cells isolated 12 h after IM revealed significant downregulation of extracellular matrix-related genes (Figure 4E). Decreased ZO-1 (a prominent tight junction protein) indicated compromised tight junctions among epithelial cells in POI mice (Figure 4F). The released granzyme B area surrounding CD8αβ^+^ IELs was indicated by IF. The line-scan profile suggested that extracellular granzyme B signal disrupts ZO-1 *in vivo* (Figure S2D). Compared to CD8αβ^+^ IELs, *P2xr7*^−/−^ CD8αβ^+^ IELs released less granzyme B into the extracellular matrix (Figure 4G) and induced less ZO-1 degradation (Figure 4H). Granzyme B protein directly degraded ZO-1 of MODE-K cells (an immortalized murine small intestinal epithelial cell line) *in vitro,* independent of perforin (Figure S2E). Flow cytometry showed that *P2xr7*^−/−^ CD8αβ^+^ IELs induce less ECC shedding (Figure 4I), and IF indicated less ECC loss induced by *P2xr7*^−/−^ CD8αβ^+^ IELs (Figure 4J). Moreover, the loss of ECCs in POI could be diminished by injection of granzyme B inhibitor Z-AAD-CMK (Figure 4K).

## Discussion

In this study, we discovered that IM surgery significantly increased activated CD8αβ^+^ IELs and their granzyme B production. The damage-induced eATP promoted granzyme B production by CD8αβ^+^ IELs via P2X7R signaling. The granzyme B derived from CD8αβ^+^ IELs exacerbated ECC loss by degrading tight junctions.

The well-established immunological mechanism of POI involves the activation of intestinal macrophages, which triggers to cytokine-driven signaling networks. Activated macrophages upregulate the expression of iNOS which is known to produce NO, thereby impacting the GI-motility.^25^^,^^26^ A 2010 study demonstrated that IL-12 stimulates intestinal memory T helper 1 cells, driving their migration and infiltration into non-manipulated intestinal tissue.^27^ These cells release cytokines that promote inflammation. Additionally, aggregation of intestinal lymphocytes can be detected in the duodenum of patients with functional dyspepsia.^28^ Intestinal CD8^+^ T cells are reported to regulate inflammation through multiple cytokines in inflammatory bowel disease.^29^ Collectively, these observations imply a potential role for lymphocytes in the etiology of surgically induced intestinal inflammation, including POI.

Our research discovered the activation and granzyme B production of CD8αβ^+^ IELs after IM. The cytotoxicity of CD8^+^ IELs is tightly regulated by epithelial cells to avoid auto-aggressive damage.^30^^,^^31^^,^^32^^,^^33^ Surgical manipulation may detach CD8^+^ IELs from inhibitory signals. Additionally, DAMPs or PAMPs activate CD8^+^ IELs, causing nonspecific tissue damage. In the intestinal ischemia-reperfusion model, necrotic cell-released cytoplasm forms the Gruenhagen space beneath the epithelium.^34^ This could lead to unintended CD8αβ^+^ IEL detachment and activation.

Purinergic signaling is a highly conserved pathway that plays a critical role in immune regulation. This pathway is stimulated by various purine derivatives, including ATP, ADP, and adenosine, with the purinergic receptor P2 family expressed on the surfaces of diverse immune cells. Significantly, the ion-channel receptor P2X7R demonstrates the broadest expression profile of all P2 receptors.^35^ In homeostasis, eATP levels are typically undetectable. However, during stress, inflammation or damage, ATP is released by both necrotic and apoptotic cells, as well as by inflamed or damaged tissues.^14^ The eATP concentrations may increase to micromolar levels.^36^ Consistent with prior research, we detected elevated eATP levels in PLF during POI.^37^ P2X2R signaling has been reported to trigger inflammation in enteric glia cells during POI.^37^ Moreover, administration of the P2X7R inhibitor A438079 has shown promise in alleviating GI-motility impairment in POI mice.^38^

Our data suggested a novel pathway in which eATP activated CD8αβ^+^ IELs and facilitated the granzyme B production. eATP has been proven to promote apoptosis of melanocytes via inflammasome and enhance the chemotaxis and the activation of CD8^+^ T cells by CXCL9^+^ cells indirectly.^39^ In addition to the activation, we observed ATP-induced cell death (AICD) under high eATP levels.^40^ Specifically, the dose-dependent increase in cell death at 100 μM ATP likely represents the functional transition of P2X7R into its “large-pore” state, which allows the passage of larger hydrophilic molecules and leads to lytic cell death.^41^ This fully aligns with the resistance of *P2rx7*^−/−^IELs to eATP-induced cytotoxicity *in vitro*. Additionally, Pannexin-1, upregulated in our study, may contribute to increased eATP levels as an ATP efflux channel. P2X7R is known to form a positive feedback loop with Pannexin-1, resulting in elevated eATP levels.^17^^,^^42^ Thus, investigating potential synergies between P2X7R and Pannexin-1 requires further research.

ECCs, a subset of differentiated gut epithelial cells, synthesize over 90% of 5-HT (serotonin).^43^^,^^44^ 5-HT plays a pivotal role in regulating GI motility and inflammation.^45^^,^^46^^,^^47^ Mechanosensitive Piezo1 stimulates ECCs, enhancing 5-HT secretion to coordinate GI motility. Additionally, the activated TRPA1, a chemoreceptor facing the lumen, facilitates 5-HT release. Mice with defective TRPA1 show pronounced GI motility impairment,^48^ highlighting ECCs’ role in food transfer during homeostasis.^49^^,^^50^^,^^51^ Furthermore, drugs such as Mosapride and Prucalopride (5-HT receptor 4 agonists) used to treat chronic constipation have been reported to improve GI motility in POI patients.^52^^,^^53^ Our data from the 5-HTP gavage assay also suggest the potential of 5-HTP treatment for POI patients. Moreover, our histological evaluation and CD8β^+^ T cell depletion assays confirmed that TPH2+ enteric neurons were not altered post-surgery. However, because eATP signaling and granzyme B release occur in a complex sterile inflammatory environment, potential direct interactions between CD8β^+^ T cells and other enteric neurons system (ENS) elements, including enteric glial cells, warrant further investigation. Future studies evaluating non-lethal functional injuries, synaptic dysfunctions, or glial-immune networks will be essential to fully clarify how deep tissue neuroinflammation intersects with the mucosal ECC-shedding axis.

IELs facilitate the shedding of epithelial cells during infection via the CD103-E-canherin pathway.^54^^,^^55^ Our data revealed the elevated CD103 on CD8αβ^+^ IELs as well as granzyme B after IM. The granzyme B is typically considered to be released via immune synapse and works in coordination with perforin.^56^ Several studies confirm extracellular granzyme B’s collagenase activity, degrading tight junction proteins between the extracellular matrix and intestinal epithelial cells *in vivo* and *in vitro.*^57^^,^^58^ Granzyme B promotes shedding of epithelial cells independent of perforin.^54^^,^^57^ Our findings elucidated that granzyme B potentially promoted the shedding of ECCs by impacting the tight-junction integrity.

During POI, numerous CD45^+^ immune cells infiltrate the intestine. Interestingly, compared to CD45^+^ immune cells, more CD45^−^ intestinal cells shed from the intestines of POI mice during IEL isolation. The granzyme B inhibitor assay suggested granzyme B and other proteinase disrupted the tight-junction integrity or the extracellular matrix. The granzyme B inhibitor VTI-1002 shows promise in clinical research for acute inflammatory injuries.^59^ Thus, we hypothesize the potential therapeutic effect of VTI-1002 in human POI. However, careful consideration is required to balance GI-motility and bacterial clearance, alongside appropriate management of necrotic tissue.

Notably, while CD8αβ^+^ IEL depletion mitigated terminal ECC loss, it failed to reduce the early CC3 increase at 12 h post-surgery. This temporal and mechanistic discrepancy highlights a multi-phase pathological cascade in POI. Surgical trauma and acute macrophage-driven inflammation independently initiate epithelial stress and apoptotic cascade (manifested as early CC3 activation at 12 h), likely mediated by macrophage-derived pro-inflammatory cytokines.^6^ Although activated CD8αβ^+^ T cells migrate to the epithelium during this early window, they do not directly aggravate this intracellular apoptotic signaling.^27^ Instead, CD8αβ^+^ IELs primarily dictate the subsequent phase at 24 h, acting as downstream executioners. Through P2X7R-mediated release of granzyme B, these IELs degrade the tight junction protein ZO-1, physically driving the shedding and loss of the pre-injured ECCs from the mucosal barrier (Figure 4). Consequently, IEL depletion halts the terminal structural sloughing process at 24 h without reversing the inflammatory stress pathways initiated at 12 h.

In conclusion, during POI, surgery-induced eATP accumulation promotes the activation and granzyme B production of CD8αβ^+^ IELs via P2X7R signaling. Activated CD8αβ^+^ IELs contribute to ECC loss and reduce 5-HT production by releasing granzyme B, which degrades tight junctions, consequently impair the GI-motility.

### Limitations of the study

Our research has certain limitations. Firstly, although CD8αβ^+^ IELs contribute to POI, macrophage-driven inflammation remains dominant. The eATP levels detected in thePLF likely underrepresent the high, transient local concentrations present directly at the site of mechanical IM.^60^ However, during intra-abdominal inflammation, higher concentrations of small-molecule inflammatory cytokines are detected in PLF rather than in the blood.^61^^,^^62^ Nevertheless, as shown in our results, the eATP concentration in PLF can reflect the severity of surgical trauma. Further validation using pmeLUC mice could provide more precise spatial measurements, our current PLF data still effectively reflect the systemic trends of purinergic surge post-surgery.^11^^,^^63^ In addition, only male mice were used in this study, and the potential influence of sex on the results was not investigated. Future studies incorporating both sexes are warranted to determine sex-specific effect in CD8αβ^+^ IEL-mediated ECC loss. Secondly, the limited availability of resected tissue from POI patients results in a lack of direct human evidence, despite reports suggesting that samples from patients undergoing pancreaticoduodenectomy provide insights into the early-stage mechanisms.^37^ Additionally, the impacts of other factors, such as endotoxins, reactive oxygen species (ROS), and metabolic abnormalities, on the epithelium during POI remain uncertain.^64^^,^^65^^,^^66^ In light of this, we propose that CD8αβ^+^ IELs facilitate the ECC loss, further research is essential to fully elucidate the underlying mechanisms.

## Resource availability

### Lead contact

Requests for further information and resources should be directed to and will be fulfilled by the lead contact, Dan Tian (tiand@ccmu.edu.cn).

### Materials availability

This study did not generate new unique reagents.

### Data and code availability

• The raw RNA sequencing data reported in this paper have been deposited in the Genome Sequence Archive in the National Genomics Data Center, China National Center for Bioinformation/Beijing Institute of Genomics, Chinese Academy of Sciences (PRJCA044457, https://ngdc.cncb.ac.cn/bioproject/browse/PRJCA044457) and are publicly available as of the date of publication.
• This paper does not report original code.
• Any additional information required to reanalyze the data reported in this paper is available from the lead contact upon request.

## Acknowledgments

Grants from the National Key Technologies R&D Program (2015BAI13B09), Beijing Natural Science Foundation (7232035), distinguished young scholars from Beijing Friendship Hospital (yyqcjh2022-4), Clinical Center for Colorectal Cancer, Capital Medical University (1192070313), the National Natural Science Foundation of China (82374190), the R&D Program of Beijing Municipal Education Commission (KZ202210025036), Beijing Municipal Administration of Hospitals’ Ascent Plan (DFL20220103), the Youth Beijing Scholar (035), and the Beijing Nova Program (20240484501) supported this work. We are grateful to the Laboratory Animal Center, Beijing Friendship Hospital for providing animal care. We thank the Institute of Clinical Medicine, Beijing Friendship Hospital, for providing experimental instruments.

## Author contributions

All listed authors participated meaningfully in the study. They have seen and approved the submission of this manuscript. F.B. and S.H. participated in performing the research, analyzing the data, and initiating the original draft of the article. X.Y., L.W., and Y.Y. participated in performing the research. D.Z., D.T., and Z.Z. established the hypotheses, supervised the studies, analyzed the data, and co-wrote the manuscript.

## Declaration of interests

The authors declare no competing interests.

## Declaration of generative AI and AI-assisted technologies in the writing process

During the preparation of this work, the authors used Kimi and Gemini in order to improve language and readability (e.g., grammar, style, and phrasing). After using these tools, the authors reviewed and edited the content as needed and take full responsibility for the content of the publication.

## STAR★Methods

### Key resources table

**Table:** 

REAGENT or RESOURCE	SOURCE	IDENTIFIER
**Antibodies**		

PE anti-mouse CD3 antibody (clone 17A2)	Biolegend	Cat#100206; RRID: AB_312663
FITC anti-mouse CD45 antibody (clone S18009F)	Biolegend	Cat#157205; RRID: AB_2894427
Brilliant Violet 711 anti-mouse CD45 antibody (clone 30-F11)	Biolegend	Cat#103147; RRID: AB_2564383
APC anti-mouse TCRγ/δ antibody (clone GL3)	Biolegend	Cat#118116; RRID: AB_1731813
Pacific Blue anti-mouse CD4 antibody (clone GK1.5)	Biolegend	Cat#100428; RRID: AB_493647
BUV395 anti-mouse CD8a antibody (clone 53–6.7)	BD Biosciences	Cat#563786; RRID: AB_2732919
BUV737 anti-mouse CD3 antibody (clone 500A2)	BD Biosciences	Cat#741716; RRID: AB_2871089
PE anti-P2X7 Receptor (extracellular) antibody	Alomone Labs	Cat#APR-008-PE; RRID: AB_2827345
FITC anti-human/mouse Granzyme B Recombinant antibody (clone QA16A02)	Biolegend	Cat#372205; RRID: AB_2687029
PE anti-human/mouse Granzyme B Recombinant antibody (clone QA16A02)	Biolegend	Cat#372207; RRID: AB_2687031
PE anti-mouse CD8β antibody (clone YTS156.7.7)	Biolegend	Cat#126608; RRID: AB_961300
InVivoMAb anti-mouse CD8β (clone 53–5.8)	BioXCell	Cat#BE0223; RRID: AB_2687706
InVivoMAb rat IgG1 isotype control, anti-horseradish peroxidase (clone HRPN)	BioXCell	Cat#BE0088; RRID: AB_1107775
Goat anti-serotonin (5-HT) antibody	Abcam	Cat#ab66047; RRID: AB_1142794
Rabbit anti-ZO-1 antibody	Abcam	Cat#ab216880; RRID: AB_2909434
Rabbit anti-Granzyme B antibody	Abcam	Cat#ab53097; RRID: AB_2114427
Rabbit anti-Cleaved Caspase-3 (Asp175) antibody	Cell Signaling Technology	Cat#9661T; RRID: AB_2341188
Rabbit anti-Cleaved Caspase-3 antibody	CST	Cat#9661T; RRID: AB_2341188
Goat anti-ZO-1 antibody	Abcam	Cat#ab190085; RRID: AB_2890613
Rabbit Anti-TPH2 antibody	Abcam	Cat# ab184505; RRID: AB_2892828
Brilliant Violet 785™ anti-mouse CD44 Antibody (clone IM7)	Biolegend	Cat#103059; RRID: AB_2571953
Brilliant Violet 421™ anti-mouse CD69 Antibody (clone H1.2F3)	Biolegend	Cat#104545; RRID: AB_2686969
PerCP/Cyanine5.5 anti-mouse CD103 Antibody (clone 2E7)	Biolegend	Cat#121416; RRID: AB_2128621
PE anti-mouse PD-1 Antibody (clone 29F.1A12)	Biolegend	Cat#135205; RRID: AB_1877232
Donkey anti-Rat IgG (H + L) Secondary Antibody, Alexa Fluor 488	Thermo Fisher Scientific	Cat#A-21208; RRID: AB_2535794
Donkey anti-Rat IgG (H + L) Secondary Antibody, Alexa Fluor 647	Thermo Fisher Scientific	Cat#A-31571; RRID: AB_162542
Donkey anti-Goat IgG (H + L) Secondary Antibody, Alexa Fluor 488	Thermo Fisher Scientific	Cat#A-11055; RRID: AB_2534102
Donkey anti-Rabbit IgG (H + L) Secondary Antibody, Alexa Fluor 488	Thermo Fisher Scientific	Cat#A-21206; RRID: AB_2535792
Donkey anti-Rabbit IgG (H + L) Secondary Antibody, Alexa Fluor 546	Thermo Fisher Scientific	Cat#A-10040; RRID: AB_2534016
Donkey anti-Rabbit IgG (H + L) Secondary Antibody, Alexa Fluor 555	Thermo Fisher Scientific	Cat#A-31572; RRID: AB_162543
PE anti-mouse TER-119 antibody (clone TER-119)	Biolegend	Cat#116208; RRID: AB_313709
PE anti-mouse CD25 antibody (clone PC61)	Biolegend	Cat#102008; RRID: AB_312857
PE anti-mouse CD19 antibody (clone 6D5)	Biolegend	Cat#115508; RRID: AB_313643
PE anti-mouse CD4 antibody (clone GK1.5)	Biolegend	Cat#100408; RRID: AB_312693
PE anti-mouse NK-1.1 antibody (clone PK136)	Biolegend	Cat#108708; RRID: AB_313395
PE anti-mouse/human CD11b antibody (clone M1/70)	Biolegend	Cat#101208; RRID: AB_312791
PE anti-mouse TCRγ/δ antibody (clone GL3)	Biolegend	Cat#118108; RRID: AB_313832

**Chemicals, peptides, and recombinant proteins**		

Tribromoethanol (Avertin)	Sigma-Aldrich	Cat#T48402
FITC-dextran, 70 kDa (FD70)	Sigma-Aldrich	Cat#FD70-100 MG
DPBS (Ca^2+^/Mg^2+^-free)	Corning	Cat#21–031-CVR
HBSS (Ca^2+^/Mg^2+^-free)	Corning	Cat#99–597-CM
Dithiothreitol (DTT)	Sigma-Aldrich	Cat#43816-10 ML
EDTA 0.5 M	Thermo Fisher Scientific	Cat#912504
Collagenase D	Roche	Cat#11088866001
DNase I	Sigma-Aldrich	Cat#DN25-100 MG
HEPES buffer (1 M)	Thermo Fisher Scientific (Gibco)	Cat#15630106
Percoll	Sigma-Aldrich	Cat#P4937
Bovine Serum Albumin (BSA)	Sigma-Aldrich	Cat#A7030
ATP (adenosine 5′-triphosphate)	Sigma-Aldrich	Cat#11140965001
ATPγS (adenosine 5′-[γ-thio]triphosphate tetralithium salt)	Sigma-Aldrich	Cat#A1388
RNALater	Sigma-Aldrich	Cat#R0901
TRIzol Reagent	Thermo Fisher Scientific	Cat#15596026
O.C.T. Compound	Sakura Finetek	Cat#4583
VECTASHIELD Antifade Mounting Medium with DAPI	Vector Laboratories	Cat#H-1200
A438079(P2X7R inhibitor)	MedChemExpress	Cat#HY-15488A
Z-AAD-CMK(Granzyme B Inhibitor II)	Calbiochem(Millipore Sigma)	Cat#368050
5-Hydroxy-L-tryptophan (5-HTP)	Sigma-Aldrich	Cat#H9772
Recombinant mouse Granzyme B protein	Sigma-Aldrich	Cat#SRP3202
DMSO	Sigma-Aldrich	Cat#D1435-500 ML
4% Formaldehyde	Guge Biological Technology	Cat#G1101
Triton X-100	Beyotime	Cat#P0096-100 mL
Fetal Bovine Serum	Sigma-Aldrich	Cat#35–076-CV
RPMI 1640 Medium (1X), with L-glutamine	Corning	Cat#10–040-CV
DMEM, high glucose (4.5 g/L), with L-glutamine and sodium pyruvate	Corning	Cat#10–013-CV
Penicillin-Streptomycin Solution, 100×	Corning	Cat#30–002-CI

**Critical commercial assays**		

Zombie Aqua Fixable Viability Kit	Biolegend	Cat#423102
Cell Activation Cocktail (with Brefeldin A)	Biolegend	Cat#423304
Cyto-Fast Fix/Perm Buffer Set	Biolegend	Cat#426803
Anti-PE MicroBeads	Miltenyi Biotec	Cat#130–048–801
EasySep Magnet	STEMCELL Technologies	Cat#18000
RNeasy Mini Kit	Qiagen	Cat#74106
PrimeScript RT reagent Kit (Perfect Real Time)	Takara Bio	Cat#RR037Q
SYBR Green PCR Master Mix	Thermo Fisher Scientific	Cat#4309155
ATP measurement Kit	Beyotime	Cat#S0027

**Deposited data**		

Bulk RNA-seq raw data(POI/Sham intestine and IEL)	This paper	Genome Sequence Archive (NGDC): PRJCA044457 https://ngdc.cncb.ac.cn/bioproject/browse/PRJCA044457

**Experimental models: Cell lines**		

The murine enterocyte cell line Mode-K	Yaji Biological	YS1673C; RRID: CVCL_B4FG

**Experimental models: Organisms/strains**		

Mouse: C57BL/6J(male, 6–8 weeks)	Vital River Laboratory Animal Technology Co.	Cat#219
Mouse: *P2rx7*^−/−^(male, 6–8 weeks)	Shanghai Model Organisms Center	Cat#NM-KO-200404
Mouse: *Rag1*^−/−^(male, 6–8 weeks)	Shanghai Model Organisms Center	Cat#NM-KO-252600

**Oligonucleotides**		

qPCR primer: mouse TPH1(F: CCA GAC ACC TGC CAT GAA CT; R: CCA TCT TGT TTG CAC AGC CC, 137 bp)	SBS Genetech	N/A
qPCR primer: mouse TPH2(F: GCA AGA CAG CGT AGT GTT CT; R: CAG TCC ACG AAG ATT TCG ACT T, 120 bp)	SBS Genetech	N/A
qPCR primer: mouse TRPA1(F: TCT TCC TGC TGG CCA TTG AG; R: AAG AGC TGA CGA GCC AGA AC, 180 bp)	SBS Genetech	N/A
qPCR primer: mouse GAPDH(F: AGG TCG GTG TGA ACG GAT TTG; R: TGT AGA CCA TGT AGT TGA GGT CA, 123 bp)	SBS Genetech	N/A

**Software and algorithms**		

FlowJo X	BD/FlowJo LLC	RRID:SCR_008520
ImageJ v1.53K	NIH	RRID:SCR_003070
GraphPad Prism v9.0	GraphPad Software	RRID:SCR_002798
DESeq2 v1.30.1	Love et al.^67^	RRID:SCR_015687
clusterProfiler v3.18.0	Yu et al.^68^	RRID:SCR_016884
ggplot2 v3.3.3	Wickham	RRID:SCR_014601
pheatmap v1.0.12	Kolde	RRID:SCR_016418
Colocalization Finder	ImageJ plugin	N/A

**Other**		

BD FACSymphony A5 Flowtytometry	BD Biosciences	N/A
Varioskan LUX Multimode Microplate Reader	Thermo Fisher Scientific	Cat#VL0000D0
Olympus IX series inverted microscope	Olympus	N/A
MACS SmartStrainer, 70 μm	Miltenyi Biotec	CAT#130–098–462
6–0 absorbable sutures	Jinhuan medical	N/A

### Experimental model and study participant details

#### Animal

Male C57BL/6J mice (Vital River Laboratory Animal Technology Co.), aged 6–8 weeks, were employed for all experiments conducted in this study. Male *P2rx7*^−/−^ mice (Shanghai Model Organisms Center), aged 6–8 weeks, were used for experiments related to P2X7R. The mice were kept in a specific pathogen-free (SPF) environment, with consistent temperature and humidity, and maintained on a 12-h light-dark cycle. Throughout the experiments, they had unrestricted access to food and water. Animal care and experimental procedures were conducted in accordance with the “Guiding Principles in the Care and Use of Animals” (China) and approved by the Ethics Review Committee of Beijing Friendship Hospital (NO. 20–2056).

Female mice were exclusively used to minimize variability. The potential impact of sex on the results remains to be determined in future studies.

#### Cell line

The murine intestinal epithelial cell line, MODE-K (Yaji Biological, Shanghai, China; CAT#YS1673C), was cultured in DMEM supplemented with 10% FBS and 1% penicillin-streptomycin at 37°C with 5% CO2.

The MODE-K cell line was authenticated by short tandem repeat (STR) profiling and matched to reference profiles in the ATCC and DSMZ databases. The cell line was tested for mycoplasma contamination by PCR and confirmed negative.

#### Ethics review

Animal care and experiment procedures were conducted in accordance with the “Guiding Principles in the Care and Use of Animals” (China) and approved by the Ethics Review Committee of Beijing Friendship Hospital (no. 20–2056).

### Method details

#### Animal model

Postoperative ileus (POI) was induced using a standardized intestinal manipulation (IM) method as previously described.^23^ Fasted mice were anesthetized with tribromoethanol (Sigma-Aldrich, CAT#T48402). To induce POI, a median laparotomy was performed, and the small intestine was gently rubbed twice with a moist cotton swab for a duration of 5 min. For the sham group, the abdomen was opened for 5 min without any manipulation. Following the procedure, the incision was closed using 6–0 sutures. To maintain body temperature, all mice were placed on a 37°C thermostat plate from the induction of anesthesia until they regained consciousness.

#### GI-transit assay

FITC-dextran (70 kDa, FD70; Sigma-Aldrich, CAT#FD70-100 MG) solution (200 μL of 5 mg/mL) was administered orally to fasted mice. After 90 min, the mice were euthanized, and the entire gastrointestinal tract, from stomach to rectum, was dissected. The gastrointestinal tract was segmented into 15 segments (from stomach to colon), numbered from 1 to 15 based on their anatomical position. Each segment was further cut into 2 mm × 3 mm pieces and placed into individual 1.5 mL EP tubes containing 1 mL of DPBS (Corning, CAT#21–031-CVR). Following centrifugation at 500 × g for 1 min, the supernatant was collected for fluorescence quantification using a fluorophotometer. Gastrointestinal transit was evaluated by calculating the geometric center (GC), using the formula provided below.

∑%FD70ineachsegment∗segmentnumber

#### Isolation of lymphocytes

Intestine: Mice were euthanized at 3 h after ATP injection. The small intestines were cleaned of fecal content, longitudinally opened, and sectioned into 1 cm pieces in ice-cold PBS. These sections were then immersed in a predigestion solution composed of Ca^2^+/Mg^2^+-free Hank’s Balanced Salt Solution (Corning, CAT#99–597-CM) supplemented with 1 mM dithiothreitol (Sigma-Aldrich, CAT#43816-10 ML) and 10 mM ethylenediaminetetraacetic acid (Thermo Fisher, CAT#912504). After filtration through a 70 μm cell strainer, the supernatant was collected to isolate the intraepithelial lymphocytes (IELs) for further experiments.

The pellet was incubated on an orbital shaker for 20 min at 37°C. Subsequently, the segments were then transferred to a digestion solution containing 500 μg/mL collagenase D (Sigma-Aldrich, CAT#11088866001), 200 μg/mL DNase I (Sigma-Aldrich, CAT#DN25-100 MG), 100 μM HEPES buffer (Thermo Fisher, CAT#15630106), and 4% FBS in RPMI 1640. After a 30-min incubation with gentle rotation, the mixture was filtered through 70 μm cell strainers. The supernatant was collected and centrifuged at 500 × g for 10 min at 20°C. The pellet was resuspended in 40% Percoll (Sigma-Aldrich, CAT#P4937) and centrifuged again, discarding the supernatant.

Mesenteric lymph nodes: Lymph nodes were placed on a 70 μm filter and ground using the end of a syringe plunger. The cell pellet was washed and resuspended in cold fluorescence-activated cell sorting (FACS) buffer (PBS with 2 μM EDTA and 0.5% bovine serum albumin (Sigma-Aldrich, CAT#A7030).

CD8β+ Lymphocytes isolation: The cells obtained above were incubated with anti-mouse CD8β (PE-conjugated YTS156.7.7, Biolegend) antibody. After washed twice with MACS buffer, the cells were incubated with anti-PE microbeads (Milteny Biotec, 130–048–801). Cell isolation was performed with EasySep Magnet (STEMCELL, CAT#18000).

#### *In vitro* activation

The cells were resuspended in the complete medium with ATP (Sigma-Aldrich, CAT#11140965001) (10 μM, 50 μM, and 100 μM)and incubated at 37°C for 30 min.

#### Flow cytometry

Zombie Aqua™ Fixable Viability Kits (Biolegend, CAT# 423102) were used to exclude dead cells. Antibody staining was performed at 4°C with fluorochrome-conjugated monoclonal antibodies according to standard methods: anti-CD3 (PE conjugated, clone 17A2), anti-CD45 (FITC conjugated, clone S18009F), anti-CD45 (BV711 conjugated, clone 30-F11), anti-TCRγδ (APC conjugated, clone GL3), anti-CD4 (Pacific Blue conjugated, clone GK1.5), anti-CD8a (BUV395 conjugated, clone 53–6.7), anti-CD3 (BUV737 conjugated, clone 500A2), anti-P2X7 (extracellular) receptor (PE conjugated, clone Q9Z1M0).

For intracellular staining, cells were incubated in a complete medium with the Cell Activation Cocktail (Biolegend, CAT#423304) at 37°C for 4 h. Cells were permeabilized with Cyto-Fast Fix/Perm Buffer Set following the manufacturer’s protocol. Fixed cells were incubated with antibody cocktail overnight at 4°C for intracellular staining: anti-Granzyme B (FITC conjugated, clone QA16A02), anti-Granzyme B (PE conjugated, clone QA16A02), Data were acquired on a FACS Symphony A5 system (BD Biosciences, San Jose, CA) and analyzed using FlowJo X (FlowJo LLC).

Anti-P2X7R receptor antibody was purchased from Alomone (Jerusalem, APR-008-PE), and the remaining antibodies, Cyto-Fast Fix/Perm Buffer Set and Zombie Aqua™ Fixable Viability kits were purchased at Biolegend.

#### Total RNA isolation

IELs or intestines pieces were immersed in RNALater (Sigma-Aldrich, CAT# R0901) and frozen at −80°C. Total RNA was isolated from tissues, using TRIzol (Thermo Fisher, CAT# Cat#15596026).

#### Quantitative PCR

Synthesis of cDNA was performed using reverse transcriptase. Gene expression was measured by real-time PCR (Thermo Fisher) with SybrGreen and Quantitative primers (Qiagen). Gene expression was normalized to the housekeeping gene GAPDH. qPCR primers were synthesized by SBS Genetech Co., Ltd., (Beijing, China).

The sequences are as follows:

qPCR primers for mouse.

TPH1 (forward 5′- CCA GAC ACC TGC CAT GAA CT-3', reverse 5′- CCA TCT TGT TTG CAC AGC CC-3', 137 bp).

TPH2 (forward 5 5′-GCA AGA CAG CGT AGT GTT CT-3′, reverse 5'- CAG TCC ACG AAG ATT TCG ACT T -3′, 120 bp).

TRPA1 (forward 5′- TCT TCC TGC TGG CCA TTG AG-3', reverse 5′- AAG AGCT GAC GAG CCA GAAC -3', 180 bp).

GAPDH (forward 5′- AGG TCG GTG TGA ACG GAT TTG-3', reverse 5′- TGT AGA CCA TGT AGT TGA GGT CA -3', 123 bp).

#### Transcriptome sequencing and data analysis of tissues or IELs

Total RNA was extracted using an RNA extraction kit (Qiagen, CAT#74106), and its quality and purity were assessed. Extracted RNA was reverse transcribed into cDNA using a reverse transcription enzyme kit (Takara Bio, CAT# RR037Q). The synthesized cDNA was fragmented and used to construct a cDNA library. The insert size of the library was detected using an Agilent 2100 (Agilent Technologies). Sequencing was performed on the Illumina platform with a PE150 strategy. The raw reads generated by Illumina sequencing were processed to obtain high-quality sequences (clean reads) by removing low-quality sequences and adapter contamination. All subsequent analyses were based on the clean reads.

The raw reads were trimmed for adapter sequences and low-quality bases using Trimmomatic v0.39. The trimmed reads were aligned to the mouse reference genome (GRCm38) using HISAT2 v2.2.1 with default parameters. The read counts for each gene were obtained using featureCounts v2.0.1 based on the Ensembl annotation (release 102). Differential expression analysis was performed using DESeq2 v1.30.1 with a false discovery rate (FDR) cutoff of 0.05. Functional enrichment analysis of differentially expressed genes was performed using clusterProfiler v3.18.0 based on Gene Ontology (GO) and Kyoto Encyclopedia of Genes and Genomes (KEGG) databases. The results were visualized using ggplot2 v3.3.3 and pheatmap v1.0.12.

#### Immunofluorescence

The intestinal tissue was rinsed with ice-cold PBS and longitudinally dissected, and then rolled into a “Swiss roll” and embedded in O.C.T. compound (Sakura Finetek, CAT# 4583) before being frozen. After sectioning the frozen tissue blocks, they were fixed with 4% formaldehyde at room temperature for 10 min, permeabilized with a 1‰ Triton X-100 solution at room temperature for another 10 min, incubated with the primary antibody solution at 4°C overnight, and then incubated with the secondary antibody solution at room temperature in the dark for 1 h. Finally, the sections were mounted with mounting medium containing DAPI (Vectashield, Vector Laboratories, CAT#H-1200). Sections were washed three times with PBS between each step.

The antibodies included in this experiment: Rat anti-mouse CD8β antibody (Lyt3.2, Bioxcell, CAT# BE0223), Goat anti-mouse serotonin antibody (Abcam, CAT# ab66047), Rabbit anti-mouse ZO-1 antibody (Abcam, CAT# ab216880), Rabbit anti-mouse granzyme B antibody (Abcam, CAT# ab53097). Rabbit anti-mouse cleaved-caspase 3 (Cell signaling, CAT#9661T), Donkey anti-rat IgG AF488 (Thermofisher, A-21208), Donkey anti-rat IgG AF647 (Thermofisher, A-31571), Donkey anti-goat IgG AF488 (Thermofisher, A-11055), Donkey anti-rabbit IgG AF488 (Thermofisher, A-21206), Donkey anti-rabbit IgG AF546 (Thermofisher, A-10040), Donkey anti-rabbit IgG AF488 (Thermofisher, A-31572).

IF images were captured using an Olympus IX10 microscope. Olympus IX1000 (Olympus) was utilized for image processing. Co-localization and semi-quantitative analyses of the images were performed using ImageJ 1.53K (National Institutes of Health). The co-localization index was calculated using the Colocalization Finder. Distinct regions of interest (ROIs) were standardized across randomly selected intact villi, and cell counts or fluorescence intensities were normalized to mucosal tissue length or area. All image analyses were performed by investigators blinded to the experimental groups using ImageJ software (v1.53K). Data for each condition was aggregated from at least 3 to 5 independent biological replicates to ensure statistical reproducibility.

#### CD8β+ IEL depletion assay

Anti-mouse CD8β antibody (Clone 53–5.8, BioXCell, CAT# BE0223) or IgG (Clone HRPN, BioXCell, CAT# BE0088) was injected i.p. Each mouse received two doses of 200 μg, spaced 48 h apart. Surgery was performed 24 h post-final injection. Subsequent assessments were conducted as previously described.

#### CD8^+^ T cell adoptive transfer

Adoptive CD8^+^ T cell transfer into *Rag1*^−/−^ mice was conducted as described previously.^69^

CD8^+^ T cells were magnetically enriched from the spleens and lymph nodes of *P2rx7*^−/−^ or WT mice. The cells were stained with an anti-mouse TER119, CD25, CD19, CD4, NK1.1, CD11b, TCR-γδ (PE-conjugated, Biolegend). Following staining, the cells were incubated with anti-PE microbeads (Miltenyi Biotec, CAT#130–048–801). Cell sorting was performed using an EasySep Magnet (STEMCELL, CAT#18000) utilizing a negative sorting strategy. Donor T cells were sex-matched to recipients. *Rag1*^−/−^ mice were injected intravenously with 1×10^7^ CD8^+^ T cells.

#### P2X7R inhibition *in vivo*

For the inhibitor group, P2X7R inhibitor A438079 (MedChemExpress, CAT# HY-15488A), was administered via intraperitoneal injection (20 mg/kg) immediately after intestinal manipulation. A438079 was dissolved in DMSO and diluted with sterilized saline. For the vehicle group, mice received an intraperitoneal injection of an equal volume of DMSO and saline. The concentration of A438079 was determined based on previous reports.^38^

#### Granzyme B inhibition *in vivo*

For inhibitor group, Granzyme B inhibitor Z-AAD-CMK (CALBIOCHEM, CAT# 368050), was administered via injection i.p. (50 μg per mouse) immediately after intestinal manipulation. Z-AAD-CMK was dissolved in DMSO and diluted with sterilized saline. The vehicle group received an intraperitoneal injection of an equal volume of DMSO and saline mixture.

#### 5-Hydroxy-L-tryptophan (5-HTP) supplement

Mice that underwent IM were orally gavaged with 5-HTP (30 mg/kg) (Sigma-Aldrich, CAT# H9772), immediately before FD70 gavage. For control group, mice were orally gavaged with an equal volume of saline. 5-HTP was dissolved in sterilized saline, with the dosage determined based on previous studies.^49^^,^^70^^,^^71^

#### Granzyme B treatment *in vitro*

The murine intestinal epithelial cell line, MODE-K (Yaji Biological), was cultured in DMEM supplemented with 10% FBS and 1% penicillin-streptomycin at 37°C with 5% CO_2_. Upon reaching 70–80% confluence on coverslips, cells were treated with active recombinant Granzyme B (GzmB) protein at gradient concentrations (0, 50, and 100 nM) for 6 h. Following treatment, cells were fixed with 4% paraformaldehyde, permeabilized with 0.1% Triton X-100. The samples were then incubated with goat anti-mouse ZO-1 antibody (Abcam, CAT#190085) overnight at 4°C, followed by a fluorophore-conjugated secondary antibody for 1 h and DAPI counterstaining.

### Quantification and statistical analysis

Statistical analyses were performed using GraphPad Prism 9.0 (San Jose, CA).Data were presented as the mean ± standard error of the mean (SEM). For comparisons between two groups, two-tailed unpaired Student’s *t* test was used. For comparisons among multiple groups (e.g., different treatment groups), one-way ANOVA followed by Tukey’s post-hoc test was used. For non-normally distributed data, the Mann-Whitney U test or Kruskal-Wallis test with Dunn’s post-hoc test was used. A *p*-value <0.05 was considered statistically significant. ∗*p* < 0.05, ∗∗*p* < 0.01, ∗∗∗*p* < 0.001, ∗∗∗∗*p* < 0.0001; ns, not significant.

### Additional resources

Not applicable.
