# Supplementary material for: P2X7R+CD8αβ+ T cells exacerbate postoperative ileus by inducing the loss of enterochromaffin cells
Source: iScience. 2026 Sep 17;29(10):117491. doi: 10.1016/j.isci.2026.117491 (PMC13602257; doi:10.1016/j.isci.2026.117491)
Supplement: Supplementary file 1 [file mmc1.pdf]

iScience, Volume *29*

Supplemental information

P2X7R+CD8$\alpha\beta^+$ T cells exacerbate postoperative ileus by inducing the loss of enterochromaffin cells

Fandi Bu, Shiyang Huang, Xiaobao Yang, Luyang Wei, Yingchi Yang, Zhongtao Zhang, Dong Zhang, and Dan Tian

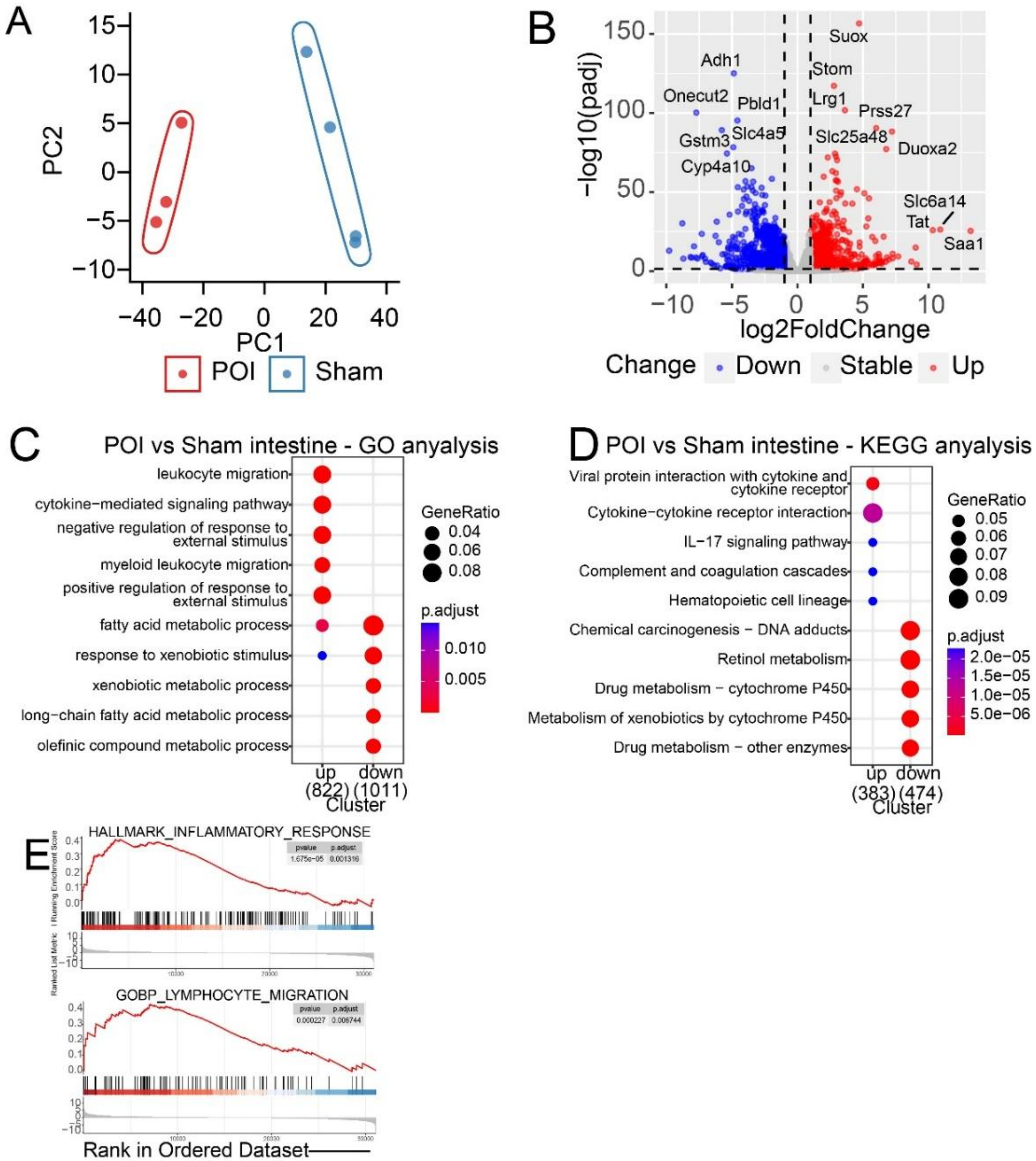

Figure S1 Transcriptome analysis of intestine for POI mice.

(A) PCA of mRNA derived from bulk RNA-seq of POI and Sham mice intestinal tissue. (B) Volcano plot showing differentially expressed genes between POI and Sham mice. (C)GO enrich analysis show the most highly enriched signal pathway in POI and Sham mice intestinal. (D) KEGG enrichment analysis of gene expression in POI mice intestine versus Sham mice intestine. (E) Selected gene sets enriched in the POI mice than those in the Sham mice were plotted.

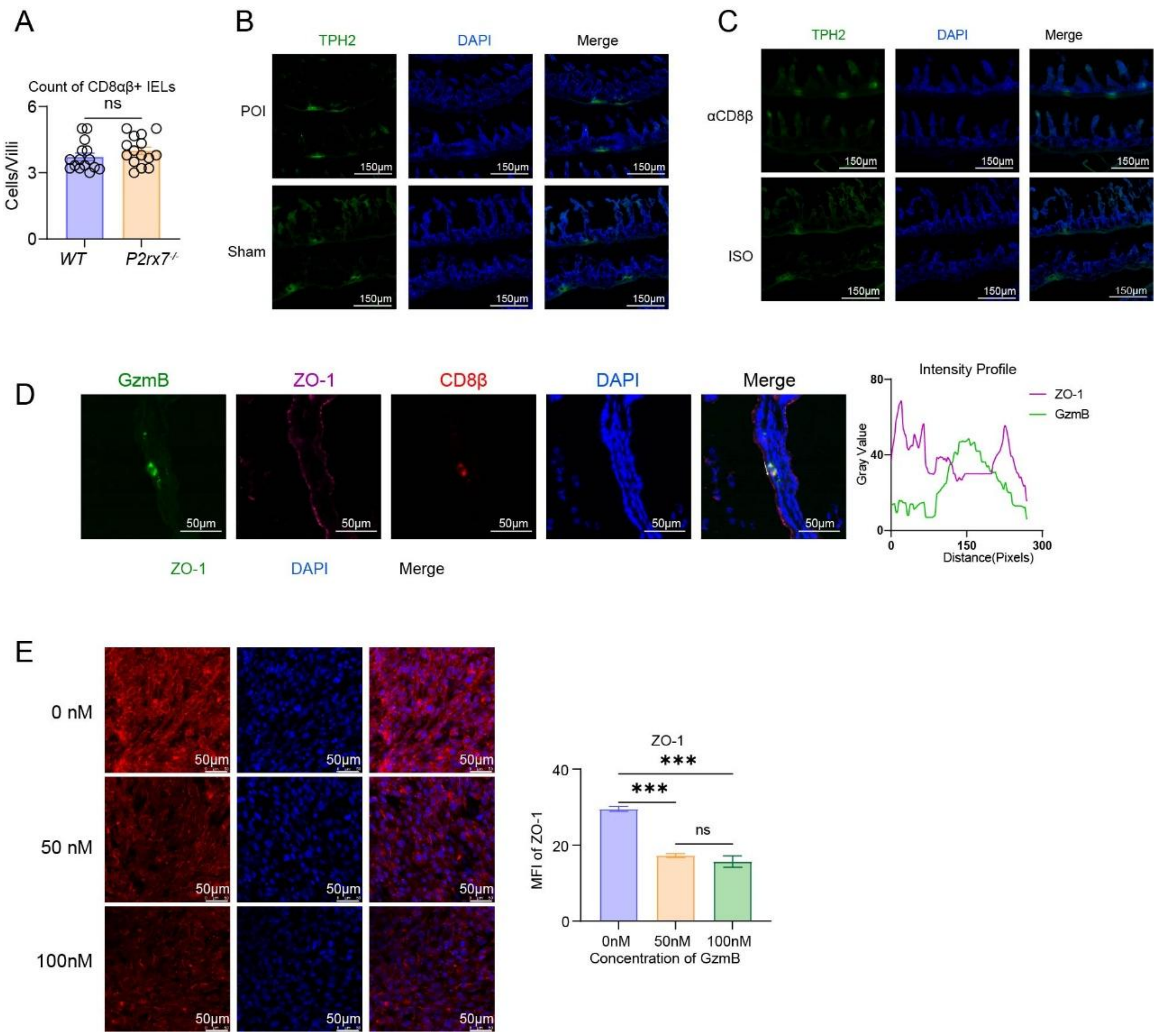

Figure S2 Granzyme B-Induced ZO-1 Degradation in POI Mice

(A) Count of CD8αβ+ IEL in small intestine after adoptive transfer in Rag1-/- mice transferred with WT and *P2rx7*$^{-/-}$ mice CD8+ T cells. n = 26 Villis, 5 mice per group. (B) Comparison of TPH2+ cells in the small intestine between POI and sham mice. Scale bar = 150 μm. (C) Comparison of TPH2+ cell in the small intestine with/without CD8β+ cells depletion. Scale bar = 150 μm. (D) Line-scan profile of GzmB and ZO-1 in POI mice intestine. Scale bar = 50 μm. (E) Immunofluorescence analysis of ZO-1 alterations in MODE-K cells following granzyme B stimulation in vitro. n=3 biological replicates. Scale bar = 150 μm. ***p < 0.001. Each column depicts the mean ± SEM
